# A case of renal solid-cystic angiomyolipoma: A case report

**DOI:** 10.1097/MD.0000000000039631

**Published:** 2024-09-13

**Authors:** Yu Shen, Zilin Wang, Zhenhua Liu

**Affiliations:** a Department of Urology, West China Hospital, Sichuan University, Chengdu, China.

**Keywords:** diagnosis, laparoscopic partial nephrectomy, renal solid-cystic angiomyolipoma

## Abstract

**Rationale:**

The different variants of renal angiomyolipoma pose diagnostic and therapeutic challenges in clinical practice. We report a rare case of renal solid-cystic angiomyolipoma, with the aim of offering new insights into the preoperative imaging assessment of renal masses.

**Patient concerns:**

A 72-year-old female was admitted to our hospital because of a solid-cystic mass discovered in her right kidney during an abdominal computed tomography scan at another hospital. Her medical history includes a 5-year history of hypertension treated with medication, as well as hepatic cysts and bilateral renal cysts.

**Diagnoses:**

The postoperative pathological diagnosis is renal solid-cystic angiomyolipoma.

**Interventions:**

The solid-cystic mass in the right kidney was surgically removed via laparoscopic partial nephrectomy under general anesthesia.

**Outcomes:**

The patient had an uneventful recovery and was discharged on the second postoperative day without complications.

**Lessons:**

Renal angiomyolipoma is usually easily distinguishable on imaging, but this case aims to alert clinicians to differentiate the rare variants of renal angiomyolipoma from other renal tumors. In the future, more cases are needed to summarize the characteristics of different variants of renal angiomyolipoma.

## 1. Introduction

Renal angiomyolipoma (RAML) is a rare benign kidney tumor typically composed of dysmorphic blood vessels, smooth muscle, and mature adipose tissue.^[1]^ It is usually asymptomatic and frequently detected incidentally during ultrasound or computed tomography (CT) scans. The management of RAML includes active surveillance, embolization, and surgical intervention depending on the risk of complications. RAML is generally identifiable on imaging due to its fatty composition; however, variants such as fat-poor or epithelioid RAML can complicate diagnosis.^[2]^ In this case report, we describe a previously unclassified renal solid-cystic angiomyolipoma, providing novel insights for preoperative image analysis. The patient provided informed consent for the publication of this case.

## 2. Case report

**Figure 1.**
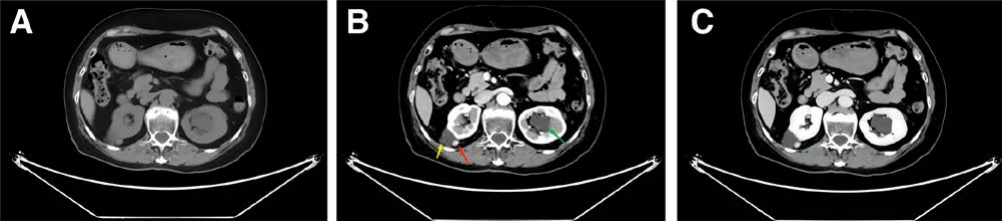
Preoperatively contrast-enhanced CT scan. (A) Plain scan, (B) arterial phase, (C) venous phase. The illustration shows the solid component (red arrow) and cystic component (yellow arrow) of the AML in the right kidney. A cyst of left kidney is also showed (green arrow). AML = angiomyolipoma, CT = computed tomography.

A 72-year-old female was hospitalized in another hospital 10 days ago due to a pulmonary infection, during which a complex space-occupying lesion in the right kidney was discovered. The patient’s height was 153 cm, and her weight was 52 kg. She had hypertension for 5 years and was well controlled by medication and had no family genetic history. Upon admission to our hospital, the patient did not exhibit low back pain or gross hematuria. Preoperative abdominal enhanced CT revealed hepatic cysts, multiple cysts in both kidneys, and a solid-cystic mass in the upper pole of the right kidney (Fig. 1). The cystic component of the mass was about 32 × 30 mm, and the solid component was located at the edge of the mass with a diameter of about 9 mm. No significant enhancement was observed in cystic component, while the solid component has a remarkably homogeneous enhancement. After discussion and communication with the patient’s family, we decided to surgically remove the solid-cystic mass. Preoperative blood tests showed no significant abnormalities.

**Figure 2.**
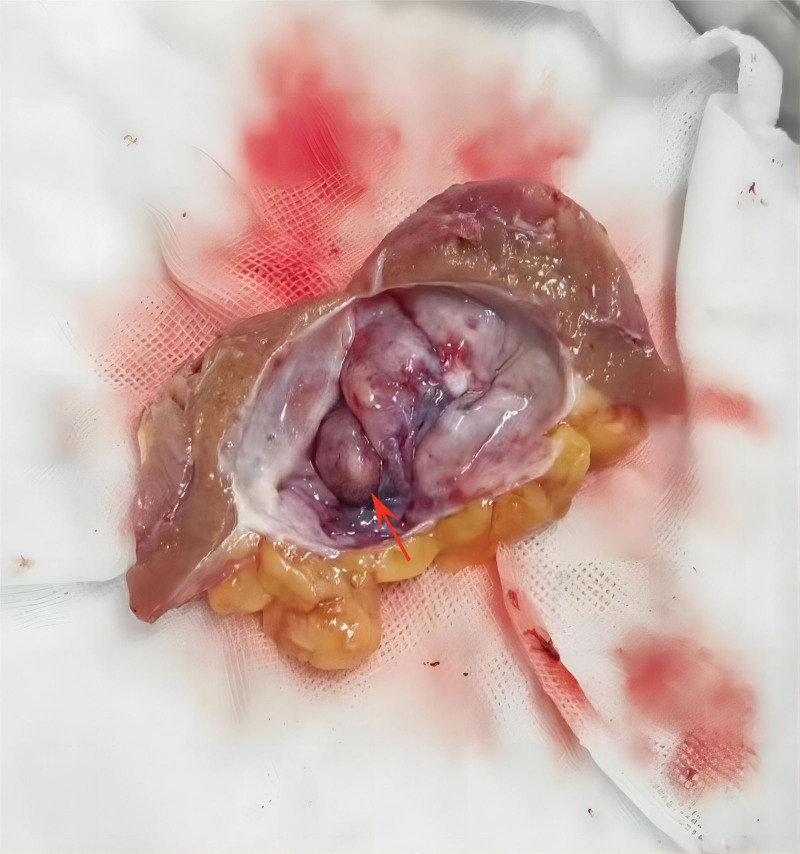
The postoperative specimen exhibits smooth cystic walls and the small solid component was indicated by the red arrow.

Given the potential for malignancy in the solid-cystic mass, laparoscopic partial nephrectomy was performed to remove the mass. The operation lasted 92 minutes and the renal artery clamping time was 20 minutes. We observed significant adhesion between the mass and the abdominal wall, the resected specimen was about 4 × 4 × 3 cm. Dissection of the specimen revealed a cystic mass measuring approximately 2.5 × 2 × 2 cm, with a smooth cyst wall and clear fluid (Fig. 2). There is a raised solid part on the cyst wall, measuring approximately 1 × 0.7 × 0.5 cm, and the cut surface of the solid component was gray-brown and fish-like. The immunohistochemical staining results were as follows: HMB-45 (partial +), SMA (+), Desmin (partial +), MART-1 (−), S100 (−), CD34 (+), CD117 (−), DOG1 (−), Calponin (+), Caldesmon (+), Collage Type Ⅳ (partial +), Ki-67 (positive rate: 1%–2%). The final diagnosis was renal solid-cystic angiomyolipoma according to the histological morphology and immunophenotype.

The patient recovered well after surgery without any complications and was discharged from hospital on the second day after surgery.

## 3. Discussion

Classic RAML is a benign neoplasm originating from mesenchymal tissues, characterized by distinctive imaging features related to its macroscopic fat content.^[3]^ The fat component of RAML is characterized by <−10 Hounsfield units in noncontrast CT, while in magnetic resonance imaging it shows high signal intensity on both T1- and T2-weighted sequences and demonstrates hypointense on fat-suppressed T1 sequences.^[4,5]^ Rare variants of RAML can complicate preoperative differential diagnosis by mimicking malignant renal lesions on imaging.^[6]^ For example, renal epithelioid angiomyolipoma, a variant classified by the World Health Organization in 2004, can be easily confused with renal cell carcinoma or fat-poor angiomyolipoma due to its lower fat content on preoperative CT or magnetic resonance imaging.^[7,8]^ Additionally, it has been reported that this variant exhibits malignant biological behavior and often leads to local progression or metastasis.^[2,8]^

In this case, we presented a histopathologically confirmed renal solid-cystic angiomyolipoma, a previously unreported classification. In order to prevent disease progression and preserve partial right renal function, we performed laparoscopic partial nephrectomy to remove the lesion. The final diagnosis was made on the basis of partial positive for HMB-45 and Desmin, positive for Calponin, and negative for S100 in immunohistochemical staining.^[9–11]^ Although pathological tests suggested that the lesion was benign, we cannot completely rule out its potential for malignant biological behavior, particularly given the invasive nature observed in other variants. The patient showed a good prognosis in the short term, but long-term follow-up was still necessary to further assess the nature of the disease. The renal solid-cystic angiomyolipoma, consisting of a large pure cyst and a small solid component, was extremely rare and has never been identified as a distinct subtype. More cases are needed to develop intervention protocols and understand the prognosis of this variant.

Classic RAML, a generally benign condition, is easily managed clinically and can often be addressed through active surveillance. For lesions larger than 4 cm or with a high risk of bleeding and rupture, prophylactic interventions such as ablation, arterial embolization, or surgery should be considered.^[1,5]^ However, diagnosing and treating the variants of RAML is more complex due to their rarity, atypical presentation, and potential for malignancy. Given the complexity, surgical removal of the lesion is feasible after evaluating potential risks until further understanding of the disease is achieved. Partial nephrectomy or radical nephrectomy can be chosen after measuring the complexity of the mass and renal function. However, this study also has some limitations. A single case cannot fully reflect the nature of renal solid-cystic angiomyolipoma, and the radiological characteristics of this lesion require more cases to be summarized.

## 4. Conclusion

Whereas RAML is relatively easy to recognize and manage, its variants are more complex and can only be diagnosed by pathological methods. We reported a renal solid-cystic angiomyolipoma that has never been classified before. This case enhances the understanding of the imaging manifestations of RAML and provides a reference for clinicians in preoperative evaluation. In the future, more cases and long-term follow-up are required to investigate this variant of RAML.

## Author contributions

**Conceptualization:** Yu Shen.

**Investigation:** Yu Shen, Zilin Wang.

**Methodology:** Yu Shen, Zhenhua Liu.

**Writing—original draft:** Yu Shen.

**Project administration:** Zhenhua Liu.

**Supervision:** Zhenhua Liu.

**Visualization:** Zhenhua Liu.

**Writing—review & editing:** Zhenhua Liu.
